# Will you swim into my parlour? *In situ* observations of Atlantic cod (*Gadus morhua*) interactions with baited pots, with implications for gear design

**DOI:** 10.7717/peerj.2953

**Published:** 2017-02-08

**Authors:** Phillip Meintzer, Philip Walsh, Brett Favaro

**Affiliations:** 1 Department of Ocean Sciences, Memorial University of Newfoundland, St. John’s, Newfoundland and Labrador, Canada; 2 Center for Sustainable Aquatic Resources, Fisheries and Marine Institute of Memorial University of Newfoundland, St. John’s, Newfoundland and Labrador, Canada

**Keywords:** Underwater video, Fisheries, Atlantic cod, Marine biology, Behaviour, Fishing gear, Behavioural ecology, Conservation, Sustainability, Newfoundland

## Abstract

Pots (also known as traps) are baited fishing gears widely used in commercial fisheries, and are being considered as a tool for harvesting Atlantic cod (*Gadus morhua*) in Newfoundland and Labrador, Canada. Pots produce lower environmental impacts than many other fishing gears, but they will only be a viable fishing strategy if they are efficient and selective at catching their target species. To study the behaviour of cod in and around pots, and how those behaviours affect pot efficiency, we used long-duration underwater video cameras to assess two models of cod pot deployed in the nearshore waters of Fogo Island, NL. We examined the number of cod that approached the pot, the number and proportion that successfully completed entries into the pot openings, and the number that exited, and related these factors to the direction of water movement. We observed very few entry attempts relative to the number of approaches by cod, and only 22% of all entry attempts were successful. We observed that 50% of approaches, 70% of entry attempts, and 73% of successful entrances occurred against the current, and 25% of cod were able to exit the pot following capture. Based on our observations, we suggest that future cod pots should have a greater number of entrances, or a mechanism to ensure that entrances rotate in line with the current, in order to maximize their catch efficiency for cod.

## Introduction

In any fishery, the type of fishing gear used influences the environmental footprint and impacts of commercial fishing operations (Chuenpagdee et al., 2003). Mobile gears, such as bottom trawls have been linked to the destruction of seafloor habitats (Freese et al., 1999) and bycatch, or the capture of non-target species (Kennelly, 1995). Bycatch has also been reported as a prominent issue with static gears such as gillnets (Northridge & United Nations Environment Programme, 1991; Regular et al., 2013) and longlines (Lewison et al., 2004; Anderson et al., 2011; Gallagher et al., 2014).

Understanding the way animals behave in response to fishing gear is one factor in assessing the gear’s impact on the environment, and understanding behaviours requires the use of underwater video cameras (Underwood, Winger & Legge, 2012). Underwater video cameras have been used to study animal behaviours near pots (also referred to as traps; Jury et al., 2001; Bacheler et al., 2013; Favaro, Duff & Côté, 2013), hooks (He, 2003), and trawls (Nguyen et al., 2014). Despite challenges such as low light levels (Underwood, Winger & Legge, 2012), cameras are beneficial because they can enable direct visual observations of the behaviours of target and non-target species within the vicinity of fishing gears. This can facilitate understanding of the interactions between marine species and fishing gears and the processes that influence the gear’s catch composition (Renchen, Pittman & Brandt, 2012).

Potting technology is a popular method of harvesting marine species in fisheries around the world (Furevik & Løkkeborg, 1994; Cole et al., 2003). Pots are a transportable, cage-like, stationary fishing gear, which typically use bait as an attractant for target species, along with retention devices to prevent the escape of caught individuals (Suuronen et al., 2012). Pots are generally selective, and are classified as a low impact fishing gear (Suuronen et al., 2012; Rotabakk et al., 2011) because they typically produce low rates of bycatch (Pol, He & Winger, 2010) and minimal impact to marine habitats. Furthermore, the stationary nature of pot-fishing typically reduces the fuel consumption of fishing vessels versus those using mobile gears (Suuronen et al., 2012). Another advantage to using pots is that trapped fish are alive and freely swimming within the pots, and are not subject to depredation, or other forms of pre-capture damage and mortality that can occur when a fish is trapped within gillnets (Walsh, Hiscock & Sullivan, 2006) and trawls (Rotabakk et al., 2011). Factors that can influence the catch rate of pots for target species include: fish density in the vicinity of gears, the feeding motivation and behaviour of the target species, the ability of fish to detect, and locate the bait within the trap, and environmental factors such as water temperature, visibility, current direction and velocity (Stoner, 2004). In Canada, pots are used to fish for several species, including spot prawns (*Pandalus platyceros*) in British Columbia (Favaro et al., 2010) and snow crabs (*Chionoecetes opilio*) in Newfoundland and Labrador (NL) (Winger & Walsh, 2011).

In NL, pots are not widely used to harvest Atlantic cod (*Gadus morhua*); however, pots are under consideration as an alternative gear on which to base a re-emergent fishery for Atlantic cod. Despite a history of intensive over-fishing (Hutchings & Myers, 1995; Hutchings & Rangeley, 2011), subsequent collapse, and continued depletion (Bradbury, 2010), the cod stock has begun to show signs of recovery, with an increase in biomass for the pre-spawning and spawning components of the “northern” cod stock detected from acoustic-trawl surveys since 2007 (Rose & Rowe, 2015). If this recovery continues, cod fishing could re-emerge as a source of income for NL communities, which would assist in the economic recovery of regions devastated by cod collapse (Schrank, 2005). However, the sustainability of this industry will depend in part on the types of gears used within the fishery, as well as management measures such as total allowable catches, quotas per fisherman, length of fishing season, the location of marine protected areas, and trap number limits per license. Traditionally, commercial-scale cod fishing has been conducted using gill-nets and bottom trawls (Hutchings & Myers, 1994). These are both efficient techniques, but they bear ecological costs, with the former producing high rates of bycatch (Northridge & United Nations Environment Programme, 1991), including marine mammals (Kastelein et al., 1995) and seabirds (Regular et al., 2013), and the latter resulting in the destruction of seafloor ecosystems (Freese et al., 1999; Thrush & Dayton, 2002), including changes to benthic species diversity and habitat loss (Thrush & Dayton, 2002). In the case of the NL cod fishery, its collapse was a product of over-exploitation (Hutchings & Myers, 1994), gear-related impacts (Milich, 1999), and environmental change (e.g., Lilly, Nakken & Brattey, 2013).

A small group of commercial fishermen on Fogo Island, NL, Canada, have been operating as a pilot-fishery for sustainable cod-fishing in NL, trialling cod pots since 2007 (Sullivan & Walsh, 2010). However, potting has not yet been adopted as a common fishing strategy with the majority of fishermen, who mainly catch fish with gillnets. Experimental cod pots have been observed to yield commercially viable catches as an alternative to gillnets along the coastline of Sweden (Königson et al., 2015), but the reluctance to switch gears in Newfoundland may be due to inefficiencies in the design of the current cod pots, such as the entrance design, and retention mechanisms, which may act as a barrier to the entry of cod (Olsen, 2014).

In this study, we used underwater cameras to assess four factors that are directly related to the efficiency of pots: the number of times that cod approached deployed pots, the number and proportion that successfully enter pots, and the number that exit the pots before they get retrieved. These parameters, taken together, describe the catch rate of a deployed pot, and problems with any one of these steps can be addressed by improved gear design, informed by underwater video (Graham, Jones & Reid, 2004).

## Materials & Methods

### Specifications of NOR pots and camera apparatus

Two styles of pots were examined: Newfoundland-style (Hereafter, NL), and Norwegian-style (NOR) pots. We tested these two models because the NL pot is currently in use by local fishermen in Newfoundland (Walsh & Sullivan, 2010), and the NOR pot is used to catch Atlantic cod in Sweden (Königson et al., 2015). Our intent was to perform a full quantitative analysis on videos collected with both pot types. However, the floating cod-end of the NL pot obstructed our camera, and therefore we had to modify the pot to provide a clear field of view. This distorted the geometry of the pot, and drastically reduced catch rates and our ability to record quantitative data. Therefore, discussion of our qualitative observations of NL pots is addressed in the supplementary materials. In this manuscript, we focus on the results we obtained using NOR pots

**Figure 1:**
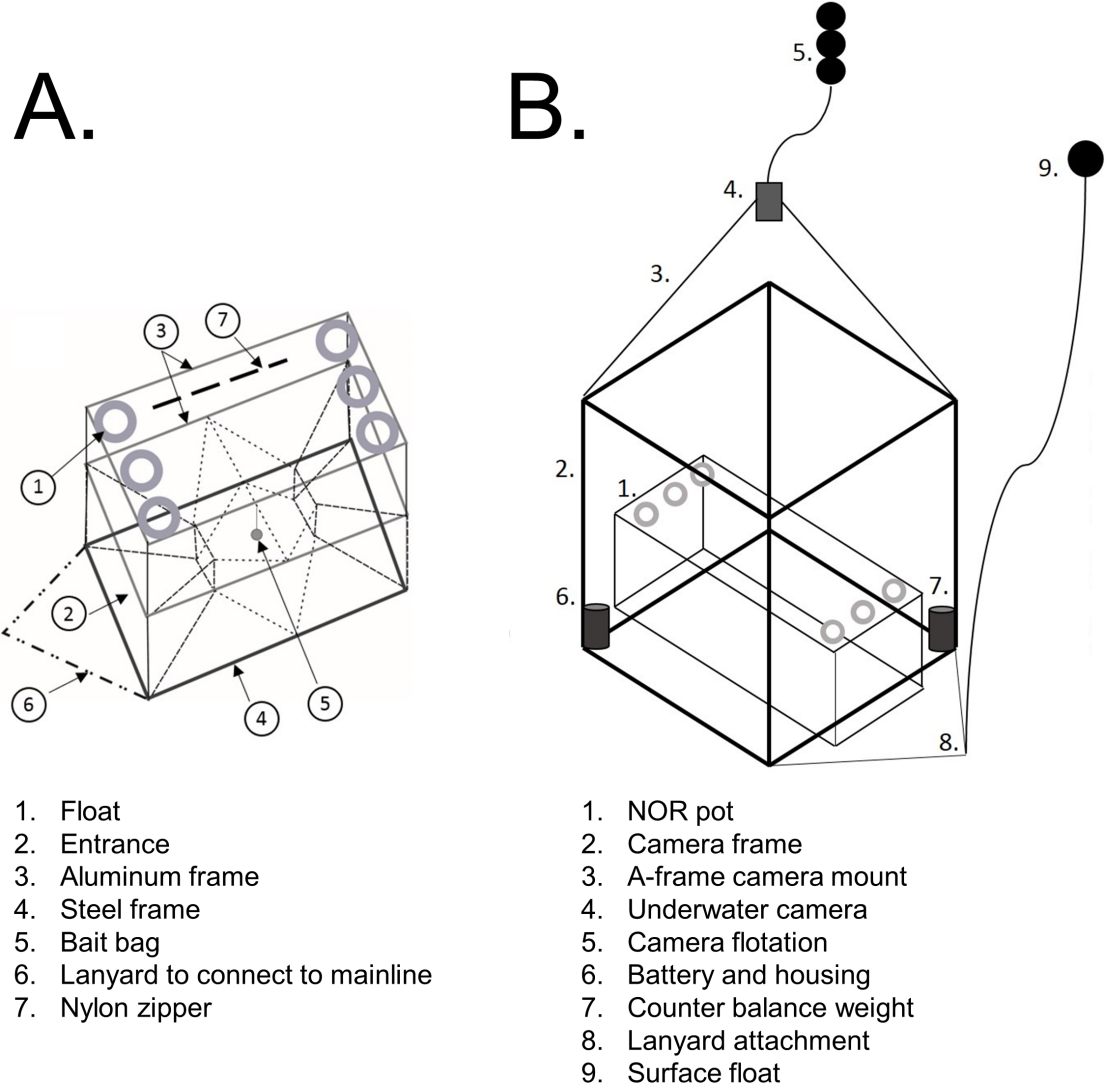
Diagrams representing the gears used during our field research. (A) is a diagram of Norwegian (NOR) pot, as it would appear deployed on the sea bottom. (B) is a diagram of the camera frame apparatus created for this study, with a NOR pot attached to the frame.

The NOR pot is similar in structure to the pots used by Furevik et al. (2008), with dimensions of 1.5 m × 1.0 m × 1.2 m (Fig. 1A). It is a two-chambered cod pot consisting of three rectangular frames in a collapsible structure. The bottom frame is made of 14 mm circular steel (to provide weight on the pot’s bottom), and the two frames above are both made of 10 mm circular aluminum. There are six floatation rings fastened to the upper mesh of the pot, which allows the pot to open vertically underwater, with the heavier frame sinking to the seafloor while the upper frame and floats extend upwards with buoyancy. The pot is divided by a mesh false bottom that extends midway through the horizontal axis of the pot, creating two-chambers. A slit in the false bottom mesh allows cod to enter the upper chamber. Zippers are present in the mesh on the side of both the lower and upper chamber to allow for removal of fish as well as easy re-baiting of the pot. The two entrances of the NOR pot face each other from opposite directions within the lower chamber, with a single bait bag suspended between them. The entrance funnels are constructed with monofilament twine.

We constructed a large aluminum camera frame for each model of pot (Fig. 1B). Both frames were rectangular prism-shaped, and were constructed of aluminum channels. The frame for the NOR pot had dimensions of 1.83 m × 1.83 m × 1.40 m made of 2.5 cm (1.0 inch) channel beams.

On top of both of these camera frames, we attached a large aluminum A-frame using rope and the under-water camera was then secured, facing downward (towards the interior of the frame) to the apex of this A-frame using metal fasteners. We attached a string of round trawl floats to the apex of the A-frame, which caused the camera to float up and above the cod pot/frame apparatus during deployment (Fig. 1B). This provided the top-down viewing angle necessary for quantitative study of potting gear (see: Favaro et al., 2012). The camera was a SubC 1-Cam Alpha+ high-definition video camera, built by SubC Imaging (Clarenville, NL, CAN). The battery for the underwater camera was stored in a plastic cylindrical housing, fastened into one of the interior corners of the camera frame, with a second housing secured to the opposite corner. This second housing contained a metal weight, used to counter-balance against the weight of the battery (Fig. 1B).

We did not use external lights, because our camera-equipped pots were set at a shallow enough depth for ambient light to illuminate the pot during the day. Artificial light has been observed to have impacts on the behaviour of fish (Dragesund, 1958; Marchesan et al., 2005; Widder et al., 2005), and previous findings suggest that cod are typically more active during the day (Løkkeborg & Fernö, 1999). Therefore, we restricted our video analysis to clips where daylight provided enough illumination for observation. As a result of this decision, the length of observable video varies for each deployment depending on time of day, water depth, and weather conditions.

### Fieldwork

**Figure 2:**
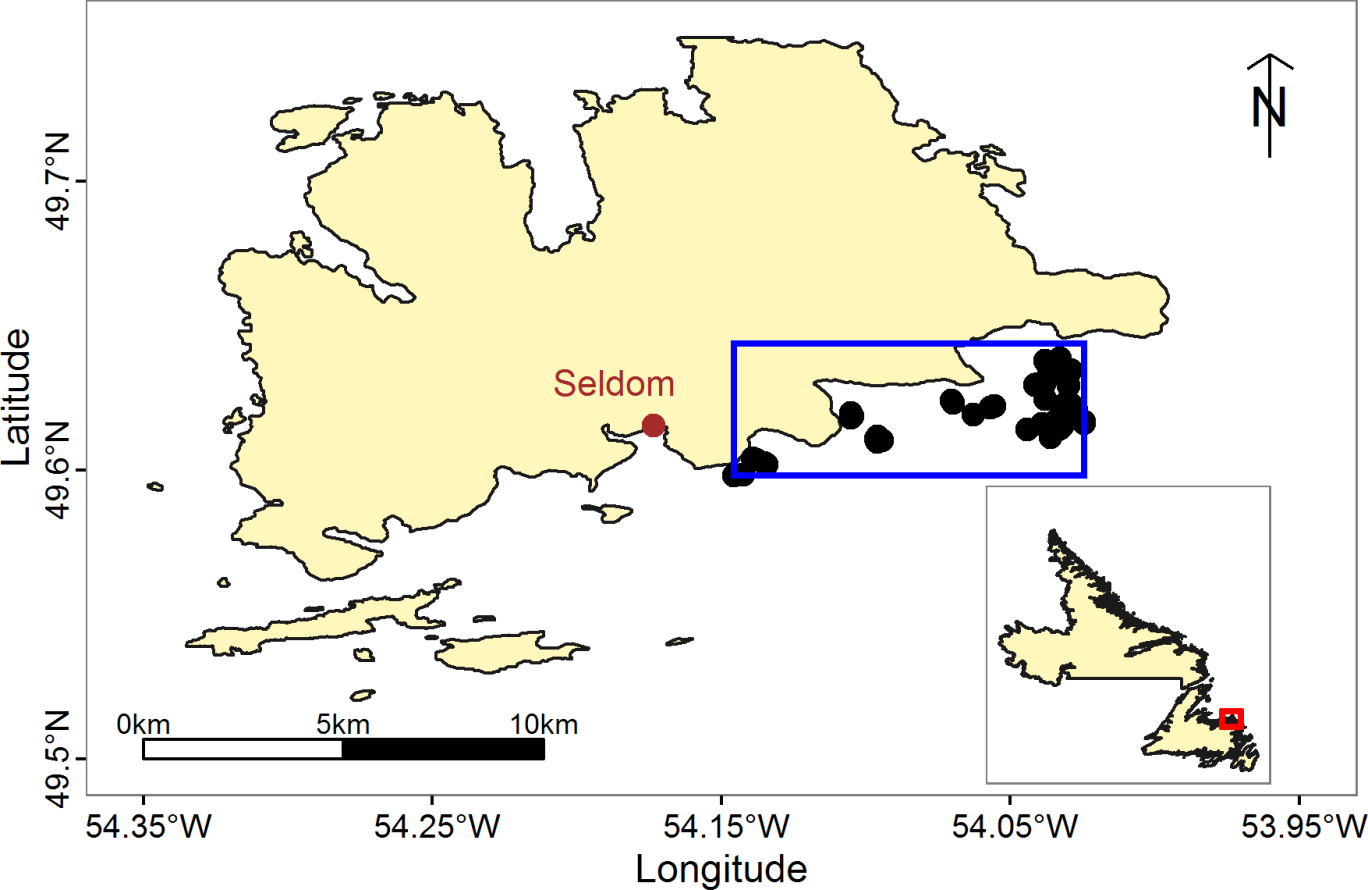
Map of our study site, off of the southern coast of Fogo Island NL. Black points indicate locations where we deployed camera-equipped pots. The blue rectangle indicates the larger fishing region of our industry partner. The red square on the inset map indicates the location of Fogo Island relative to the rest of NL. Map data from GADM database of Global Administrative Areas (http://gadm.org/).

We conducted our fieldwork in the nearshore waters, within 5.00 km (2.70 nautical miles) of southern Fogo Island, NL (Fig. 2), for a three-week period during August and September 2015. We recorded our videos during the small-scale Atlantic cod stewardship fishery operating during that same time period. Our field work was conducted on the 10.4 m (34 foot) fishing vessel *Dean & Michael*, operated by commercial fishers based in Seldom, NL. We deployed our camera-equipped pots in areas where commercial fishing experience suggested that cod density would be sufficient to support commercial fishing.

We programmed our camera to record continuously from the time of gear deployment until recovery, which was typically the following day. Soak times (i.e., the amount of time between the camera was deployed and retrieved) were as close to 24 h as possible, but varied due to logistical constraints such as weather (Table 1). Our decision to use 24-hour soak times was consistent with the soak times used by commercial fishers, and were similar to those used in previous studies conducted with gillnets (Gearin et al., 2000). Following the retrieval of our camera, we recorded the total catch (of cod and bycatch) within the camera-equipped pot, and then re-baited the pot for its next deployment using five frozen squid (*Illex illecebrosus*). Squid were used as bait based on the commercial fishing experience of our fishermen partners, as well as previous studies which demonstrated the effectiveness of this bait type (Furevik & Løkkeborg, 1994). Five frozen squid per deployment was sufficient bait for this experimental design, as previous studies have successfully captured cod using as few as three frozen squid per deployment (Furevik et al., 2008). We downloaded the videos from the camera’s onboard memory after each deployment, and connected a fully charged battery for the next deployment.

**Table 1:** Summary of camera deployments for NOR pots.

Deployment number	Pot type	Start date	Start time	End date	End time	Observed video time (mins)
1	NOR	19/08/2015	15:15:00	20/08/2015	8:46:28	281
2	NOR	20/08/2015	10:34:28	21/08/2015	12:15:13	651
3	NOR	21/08/2015	15:47:00	22/08/2015	12:51:27	555
4	NOR	22/08/2015	14:46:30	23/08/2015	12:54:10	431
5	NOR	23/08/2015	14:48:06	26/08/2015	10:50:43	950
6	NOR	26/08/2015	16:12:37	27/08/2015	12:05:35	498

To determine if the presence of our camera apparatus affected the catch rates of our pots, we compared the catch-per-deployment of camera-equipped NOR pots with the catch rates of 72 commercially fished NOR pots within the same fishing region. NOR pots with cameras were deployed one-at-a-time, while the non-camera NOR pots were fished in connected ‘fleets’ of four or five pots. We used the same type and amount of bait (five frozen squid) in these commercially fished pots as well as our camera pots. To compare the catch rates between camera and non-camera pots, we used generalized linear mixed-effects models (GLMMs; Zuur et al., 2009). We built a model that measured the impact of the fixed effect of camera presence or absence on catch-per-deployment. We included Fleet ID as a random effect to account for the fact that non-camera NOR pots were nested within fleets. The distribution of our catch data was best explained by a negative binomial distribution. Residuals met the assumptions for homogeneity, normality, and independence.

### Video analysis

At the conclusion of the field study, we watched all the videos that we recorded. Our camera provided a top-down viewing area of approximately 1.80 × 3.27 m around the NOR pot. We recorded the following quantitative parameters from each video: prevailing direction of water movement (in each one-minute segment of video), the number of each cod that approached the pot and the direction they approached from, the number and direction of cod that attempted to enter the pot, and the proportion of those entries that were successful, and the number of cod that exited the pot after entering it. We defined an approach as a cod entering the visible area of the video. Note that if a fish was to swim towards the pot, swim away, and then return to the visible area of the video, we would record this as two separate approaches. The cumulative number of successful entrances over time (minus exits) gave us the total number of cod in the pot at any given time across the deployment. After the overnight soak, we manually counted the number of cod visible in the pot to give us an estimate of the number of cod in the pot in the morning. From that point, we resumed calculating the total number of cod in the pot as a sum of the number of entries minus exits over time.

We recorded the direction of cod approaches, entry attempts, and successful entrances, in relation to the direction of water movement. We scored these factors as occurring with-current, against-current, or perpendicular to the current. For instances when an approach was made while the current direction was not clearly determinable, due to visibility, camera movement, or turbulent water movement, we excluded that approach from this part of our analysis.

We defined an entry attempt as an instance where any portion of an individual cod’s body crossed over the exterior limit of the funnel mesh for either entrance of the pot. We recorded the total number of attempts, and which entrance (with-current, against-current, or perpendicular-current) the attempt occurred at. The result of every entrance was scored as either a failed attempt, where the individual retreated out from the entrance funnel, or as a success, where the individual’s full body crossed over the ending of the interior portion of the entrance funnel mesh, and into the body of the pot. We defined a successful entrance as an instance where the whole body of an individual cod crossed over the interior limit of the funnel mesh for either entrance of the pot. We recorded the total number of successful entrances, and which entrance (with-current, against-current, or perpendicular-to-current) the success occurred at. We assessed whether there was an association between the type of interaction (approaches, entry attempts, and successful entries) and direction of water movement (against-current, with-current, and perpendicular), using a chi-squared test.

The project was reviewed and approved by Memorial University’s Institutional Animal Care Committee (Project # 15-03-BF).

## Results

### Camera impact

We found no impact of the presence of the camera on cod CPUE (GLMM: *β* =0.10, S.E. =0.21, *z* = 0.48, *p* = 0.63). The mean catch rates of cod per deployment (±1 S.E.) for NOR pots without cameras was 25 ± 1 compared to 27 ± 6 for NOR pots with a camera.

### Video analysis

We deployed our video apparatus six times with the NOR pot (Table 1). Deployment depths ranged from 28.35 to 44.99 m (mean ± 1 S.E. = 36.21 ± 2.91). Soak times ranged between 17.52 and 68.04 h (mean ± 1 S.E. =29.06 ± 7.87). Soak times did not always match video length because we were not always able to retrieve and deploy the camera frame at the same times every day due to inclement weather, and in one instance our battery did not have sufficient charge to last until retrieval. From these six deployments, we collected approximately 135 h of under-water video footage. Video recordings ranged from 18.15 to 28.12 h for NOR pots (mean ± 1 S.E. =23.39 ± 2.30). Of the 135 h of video collected, 56.10 h had sufficient ambient lighting to undergo quantitative analysis, as a result of our decision to not use supplementary illumination, and varying levels of ambient light. We analyzed all 56.10 h of observable video collected for the NOR pot.

**Figure 3:**
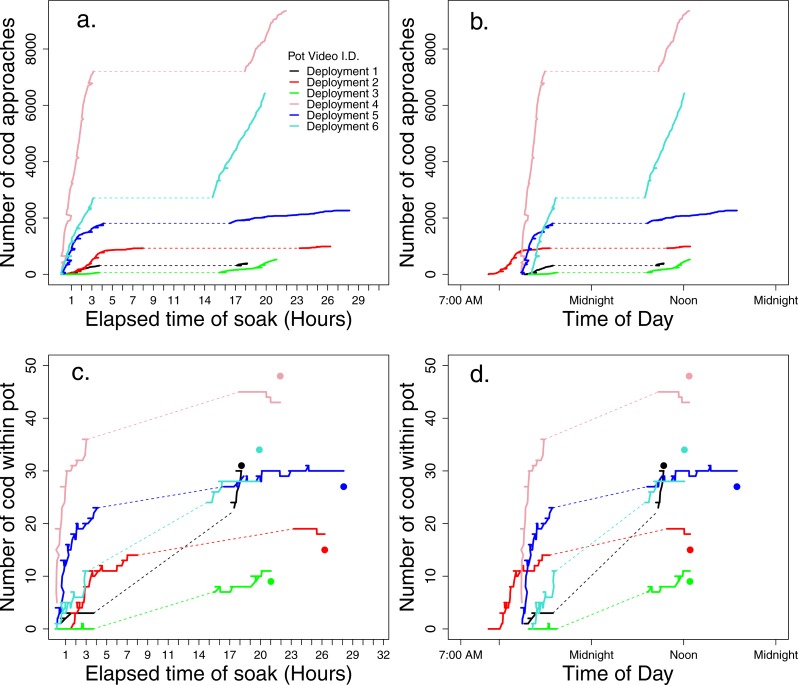
Comparison of Atlantic cod accumulation for NOR pots, over the course of both elapsed time and real time for each deployment (*N* = 6). Plots (A) and (B) display the accumulation over the elapsed soak time, whereas plots (C) and (D) display the accumulation over real time. Approaches by cod are shown in both (A) and (C), and the accumulation of cod successfully within the pot are shown in (B) and (D). Each colored line represents and individual deployment. Dashed lines represent time periods where camera footage was absent (due to low-light conditions). Coloured circles in (C) and (D) represent the final catch of each pot deployment. Lines represent observed catches, and dots represent the actual landed catch, recorded at sea, when the pot was hauled.

We observed a total of 19,940 approaches by cod across all six deployments (Table 2 and Fig. 3), and we observed between 389 and 9,349 total cod approaches (mean ± 1 S.E. = 3,323 ± 1,516) per deployment. It took 11.3 min on average for the first cod to approach a pot (*N* = 6, S.E. =8.4, range = 1–53 min; Fig. 3), and it took 51.9 min on average for the first cod to successfully enter the pot (*N* = 6, S.E. = 26.2, range = 4–157 min; Fig. 3). We observed a total of 34 cod exit the pots across all six deployments (Table 2 and Fig. 3).

**Table 2:** Summary of cod behaviour in the vicinity of NOR cod pots. Behaviours are summarized per camera-pot deployment, Deployment ID corresponds to one of our six camera-attached NOR pot deployments, approaches corresponds to the number of cod observed to enter the field of view (FOV) of the video recording, and entry attempts describes the total number of observed attempts to enter the pot. An exit describes when a cod that was already successfully caught within the pot, managed to escape the pot back into open water.

Pot type	Deployment ID	Approaches	Entry attempts	Successful attempts	Failed attempts	Exits
NOR	1	389	35	11	24	0
	2	988	71	21	50	8
	3	524	48	7	41	3
	4	9,349	187	37	150	3
	5	2,265	146	41	105	15
	6	6,425	148	20	128	5
**Total**	****	**19,940**	**635**	**137**	**498**	**34**

There were very few entry attempts relative to the number of approaches towards the pot by cod, with only 3.2% (*N* = 635) of the number of entry attempts relative to approaches (*N* = 19,940; Table 2). The proportion of entry attempts that were successful was similarly low; across six deployments, 635 cod attempted to enter, with only 137 (22%) successfully entering the pot (Table 2). Of those 137 cod that were able to successfully enter the pot, 25% (*N* = 34) were able to exit prior to retrieving the gear (Table 2).

**Figure 4:**
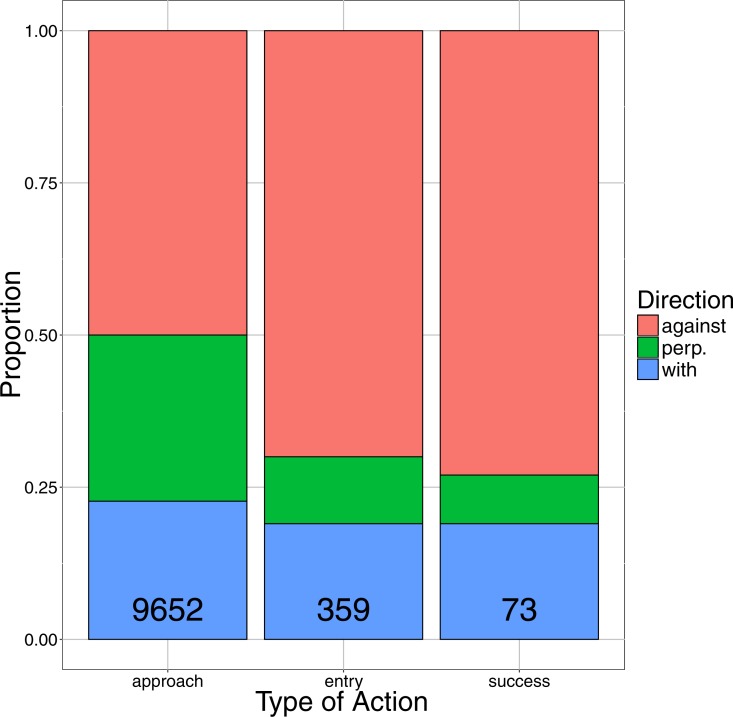
The proportions of approaches, entry attempts, and successes, occurring from the with-current, against-current, and perpendicular-current direction for the NOR pot. Numerical values represent the total number of actions (approach, entry attempt, or success) observed.

We were able to successfully quantify the water direction for 9,652 approaches for the NOR pot (*N* = 10,288 approaches occurred during sections of video where the water direction was unable to be accurately determined due to variable currents, reduced visibility, and camera movement). A total of 50.0% (*N* = 4,821) of cod approached the pot from the down-current direction, with 27.3% (*N* = 2,639) approaching perpendicular to the pot, and 22.7% (*N* = 2,192) approaching from the upstream direction (Fig. 4). For entry attempts compared to water direction, we were able to successfully quantify the water direction for 359 entry attempts. We observed 250 entry attempts (70%) at the downstream (against-current) facing entrance, with 67 entry attempts (19%) occurring at the upstream (with-current) facing entrance, and 42 attempts (11%) occurring when the current was perpendicular to the entrances (Fig. 4). For successful entries into the pot, we were able to successfully quantify the water direction for 73 successful entries. We observed 53 successful entry attempts (73%) at the downstream (against-current) facing entrance, 14 successful entry attempts (19%) at the upstream (with-current) facing entrance, and six successful entrances (8%) occurring when the current was perpendicular to the entrances (Fig. 4). Through our chi-squared test, we rejected the null hypothesis that there was no relationship between the count of approaches, entry attempts, and successful entries and water direction (*χ*^2^ = 69.9, *df* = 4, *p* < 0.001).

We observed only three non-target species approach the NOR pot across all six deployments. The non-target species most observed was toad crab (*Hyas araneus*) which approached the pot 154 times total across all six deployments. We observed between 0 and 66 toad crab approach the NOR pot per deployment (mean ± 1 S.E. =25.67 ± 11.95), with only five individual toad crab successfully entering the NOR pot across all six deployments. We saw 30 approaches by short horn sculpin (*Myoxocephalus scorpius*), and two approaches by a species of flatfish (order Pleuronectiformes). Neither of these successfully entered the pot.

## Discussion

Although NOR pots were able to successfully capture cod, the majority of entry attempts were not successful. The low proportion of successful entries into the NOR pot appears to be a result of the direction of water flow relative to the pot orientation. We observed that a greater number of cod approached the NOR pot from the down-current direction. In addition, a greater number of entry attempts and successful entrances occurred at the down-current facing entrance. These observations are consistent with previous research which has described that cod will approach bait from the down-current direction (Løkkeborg, Bjordal & Ferno, 1989). We also observed many instances of individual cod or groups of cod approaching the pots and attempting entry from the down current direction, regardless of the actual entrance location, resulting in cod attempting to push through the mesh at places where an entrance was not present. This indicates that in order for a pot to maximize its catch efficiency, at least one of the pot’s entrances should be in line with the down-current water direction, to ensure cod are able to locate the entrance. Our finding supports the logic of Scandinavian fishermen who have used floating pots that can orient in the direction of water movement (Furevik et al., 2008; Bryhn et al., 2014; Königson et al., 2015). Alternatively, future designs could feature entrances on all sides of the pot so that at least one will line up with the down-current direction. One limitation of this study design was that the 19,940 approaches by cod did not likely represent 19,940 individual fish—since each approach by an individual fish that repeatedly re-enters the visible frame would be counted separately.

We found that cod were able to exit pots, but that exits were uncommon. These exits were observed as early as eight minutes following the start of a deployment, indicating that cod are able to locate the exits to the pots earlier than expected based on previous studies (Königson et al., 2015). One issue that needs to be addressed with the NOR pot to reduce exits is the distance separating the two entrance funnels. The small size of the pot in conjunction with the entrances directly opposing one another results in cases where cod successfully enter the pot through one entrance, but then swim right through and exit via the opposite opening. The majority of cod that successfully entered the NOR pot swam into the pot’s upper chamber, and did not generally return to the bottom chamber. The majority of cod that escaped did so before entering the upper chamber.

For the majority of our video deployments, we also observed that there were fewer successful entry attempts made by cod following the overnight period (Fig. 3). We propose two non-exclusive hypotheses for this observation. First, the bait may be less attractive as time goes on, either because its mass is reducing due to consumption, or because of bait plume depletion. Previous literature has shown that high release rates of attractants from bait is required to attract fish to fishing gears (Løkkeborg & Johannessen, 1992), and this may indicate why fewer successes are observed following the overnight period in our videos. Second, the pot may approach saturation in the early morning period e.g., Ovegård et al. (2011). However, we find the second hypothesis un-compelling because our six pots—which were effectively identical—appeared to ‘saturate’ at very different densities.

From our video observations, typically, following successfully entry into the pot, cod individuals would interact with the bait bag, and then swim upwards and enter the upper chamber of the pot. Once inside the upper chamber of the pot, the majority of fish begin exhibiting positive rheotaxis. Occasionally, an individual may exhibit escape behaviours once inside the pot, indicated by excited movements and attempting to press through the mesh walls of the pot with their snouts. This behaviour has been observed in previous research (Renchen, Pittman & Brandt, 2012), and could be motivated by cannibalistic behaviours between trapped cod individuals (Bogstad et al., 1994), however over time these individuals eventually resume rheotaxis, and for videos recorded in the morning, following an overnight soak, the majority of all fish within the pot were exhibiting rheotaxis simultaneously. For undersized or juvenile cod who become trapped in pots, larger mesh escape panels can be installed to allow for escape, reducing undesirable catches for the fishermen (Ovegård et al., 2011; Königson et al., 2015).

Very few non-target species approached our deployed pots, with only 186 total approaches observed for toad crab, sculpin and flatfish combined, across all six deployments, with only five toad crab successfully entering the pot. We saw no instances of non-caught individuals becoming trapped or entwined in the mesh of the pots. This stands in contrast to traditional commercial cod fishing gears, such as gillnets, which can substantially reduce seabird populations as a result of bycatch (Regular et al., 2013), and which can ensnare substantial numbers of marine mammals as well (Kastelein et al., 1995; Read, Drinker & Northridge, 2006). Toad crab made up the largest proportion of bycatch for the NOR pots, and minimizing this bycatch could be a goal for future improvements to the design of this gear. An alternative strategy is to acknowledge this bycatch in the conditions of fishing licenses, require fishermen to land it, and manage as a multispecies fishery e.g., Gislason et al. (2000) and Grafton, Nelson & Turris (2005). The presence, and orientation of the two chambers within the NOR pot could even allow for multi-species targeting, with shellfish accumulating in the lower chamber, and cod within the upper chamber, if a multi-species fishery were established.

### Implications for pot design

We found that NOR pots (when baited with squid) are successful at attracting a large number of cod towards the vicinity of the pot, and that the pots are able to successfully retain the vast majority of their caught cod, with only a small proportion of cod escaping. However, the proportion of cod within the vicinity of the pot that attempted and successfully completed entry attempts could be improved. Therefore, we suggest that future cod pot designs should feature an increased number of entrances, or a mechanism allowing for the orientation of entrances in-line with the downstream current direction, in order to increase the number of entry attempts and successful entries by cod.

At the moment, the financial viability of cod pots as the primary harvesting tool for cod fishermen in NL is uncertain. The current cod pots we tested were prototypes built for research purposes. Determining the large scale viability of pots requires data on many variables, including the initial cost for purchasing a fleet of commercial cod pots, the average fuel costs to harvest a commercial fleet of pots, the average mass of cod collected from a fleet of pots, and the sale price of Atlantic cod paid to the fishermen (which is variable depending on the quality of the caught cod).

Pots are generally considered a low-impact fishing gear, because of their reduced bycatch, live discards, and reduced fuel consumption (Suuronen et al., 2012). In addition to these benefits, pots have been observed to have higher discard survivability, with previously captured cod, becoming re-captured in pots following release, in successive deployments (Pol & Walsh, 2005). The greater survivability of pot caught individuals, could provide increased options to fisheries managers with regards to management decisions on the required landing of discards. Basing a resurgent cod fishery on pots therefore stands to produce conservation benefits relative to other gears. The information gained from this research indicates that NOR pots are generally well-designed for catching cod selectively, but there remains opportunity for improvement. Specifically, that the bottleneck in capture appears to occur at the entrances, and modifications to improve entry rates could greatly enhance the efficiency of this fishing gear.

##  Supplemental Information

10.7717/peerj.2953/supp-1 Data S1 Meintzer, Walsh & Favaro—Cod Pot Underwater Video Raw Data 2015

10.7717/peerj.2953/supp-2 Article S1 Supplementary Article—Methods and Results for NL Pots

10.7717/peerj.2953/supp-3 Table S1 Summary of NL pot deployments with camera Summary of camera deployments for NL pots.

10.7717/peerj.2953/supp-4 Figure S1 NL cod pot diagram Diagram representing the Newfoundland (NL) cod pot used during our field research.

10.7717/peerj.2953/supp-5 Figure S2 End effect plot In response to reviewer 2—comment [j15]. A box plot comparing the number of Atlantic cod caught per pot deployment, at different pot locations within a single fleet (string) of NOR cod pots. A distance of zero indicates the pot was located at the end of the fleet.

## Additional Information and Declarations

### Data Availability

The following information was supplied regarding data availability: Meintzer, Phillip (2016): Supplementary Video—NOR Cod Pot Underwater 2015.mp4. figshare. https://dx.doi.org/10.6084/m9.figshare.4300070.v1.
