# Supplementary material for: Will you swim into my parlour? In situ observations of Atlantic cod (Gadus morhua) interactions with baited pots, with implications for gear design
Source: PeerJ. 2017 Feb 8;5:e2953. doi: 10.7717/peerj.2953 (PMC5301977; doi:10.7717/peerj.2953)
Supplement: Article S1 [file peerj-05-2953-s002.docx]

**Methods**

*Specifications for the NL cod pot*

The NL pot is large (2 m x 2 m x 1 m) and comprises a frame built of round reinforcing steel, covered by polyethylene mesh (100 mm x 3 mm; Walsh & Hiscock, 2005; Supplemental Figure S1). The NL pot has two offset entrance funnels, typically constructed with white nylon mesh. The interior end of these entrance funnels contains a metal retention device known as a trigger, which uses long metal finger-like projections to allow one-way movement into the pot, and to prevent escape. At the top of the pot, there is a large expandable mesh roof, known as a cod-end, which is supported by floats that extend upward during the pot’s deployment.

We constructed a large aluminum camera frame for each model of pot (Figure 1B). Both frames were rectangular prism-shaped, and were constructed of aluminum channels. For the NL pot, the frame dimensions were 2.44 m x 1.83 m x 1.22 m, using square aluminum channel beams 3.8 cm (1.5 inches) in width.

It was our intent to perform a full quantitative analysis on videos collected with both pot types. However, the floating cod-end of the NL pot obstructed our camera, and therefore we had to modify the pot to provide a clear field of view. This distorted the geometry of the pot, and drastically reduced catch rates relative to NL pots without cameras. Therefore, we limited our analysis of NL pots to qualitative observations only, noting the behaviours of cod and other species in and around pots. For the NOR pot, we recorded both qualitative and quantitative data.

**Results:**

We deployed our video apparatus four times with the NL pot (Supplementary Table 1). From these four deployments, we collected approximately 79 hours of under-water video footage. Video recordings ranged from 5.63 to 30.73 hours for NL pots (mean ± 1 S.E. = 19.66 ± 5.51). Of the 79 hours collected, 30.25 hours had sufficient ambient lighting to undergo quantitative analysis, as a result of our decision to not use supplementary illumination, and varying levels of ambient light. We analyzed all 30.25 hours of observable video collected for the NL pot.

Although our apparatus made quantitative analysis of the NL pots impossible, we were able to make qualitative observations of its performance. We found that cod typically attempted to enter the NL pot through the mesh at heights similar to the height of the bait bag, inappropriate for successful entry. This could indicate that the bait bag needs to be closer to the bottom of the pot, or at least level with the entrance funnel height. The inappropriate height of the bait bag might not only affect entrance attempts, but could also influence the detection of the bait bag and bait plume, which may at least partially explain why we observed fewer cod approaching the NL pots, because correct bait plume orientation with pot entrance funnels and current direction are important factors influencing a pots catchability (Pol, He & Winger, 2010). We have also observed many cod entry attempts deterred by the presence of the metal triggers, similar to the observations made by Olsen, 2014. Individual cod change direction and exit the entrance funnels following contact with the triggers, however small cod individuals pass between the triggers’ rods without contact.

**Supplementary Tables**

Supplementary Table 1. Summary of camera deployments for NL pots.

| Deployment number | Pot type | Start date | Start time | End date | End time | Observed video time (mins) |
| --- | --- | --- | --- | --- | --- | --- |
| 7 | NL | 29/08/2015 | 12:39:30 | 30/08/2015 | 14:17:51 | 795 |
| 8 | NL | 30/08/2015 | 16:32:47 | 31/08/2015 | 9:08:00 | 109 |
| 9 | NL | 31/08/2015 | 10:23:00 | 1/9/2015 | 8:40:32 | 338 |
| 10 | NL | 1/9/2015 | 10:17:38 | 3/9/2015 | 7:16:37 | 573 |
