# Supplementary material for: Will you swim into my parlour? In situ observations of Atlantic cod (Gadus morhua) interactions with baited pots, with implications for gear design
Source: PeerJ. 2017 Feb 8;5:e2953. doi: 10.7717/peerj.2953 (PMC5301977; doi:10.7717/peerj.2953)
Supplement: Table S1 — Summary of camera deployments for NL pots. [file peerj-05-2953-s003.docx]

**Tables**

Table 1. Summary of camera deployments for NL pots.

| Deployment number | Pot type | Start date | Start time | End date | End time | Observed video time (mins) |
| --- | --- | --- | --- | --- | --- | --- |
| 7 | NL | 29/08/2015 | 12:39:30 | 30/08/2015 | 14:17:51 | 795 |
| 8 | NL | 30/08/2015 | 16:32:47 | 31/08/2015 | 9:08:00 | 109 |
| 9 | NL | 31/08/2015 | 10:23:00 | 1/9/2015 | 8:40:32 | 338 |
| 10 | NL | 1/9/2015 | 10:17:38 | 3/9/2015 | 7:16:37 | 573 |
