# Supplementary figures and images for: Will you swim into my parlour? In situ observations of Atlantic cod (Gadus morhua) interactions with baited pots, with implications for gear design
Source: PeerJ. 2017 Feb 8;5:e2953. doi: 10.7717/peerj.2953 (PMC5301977; doi:10.7717/peerj.2953)

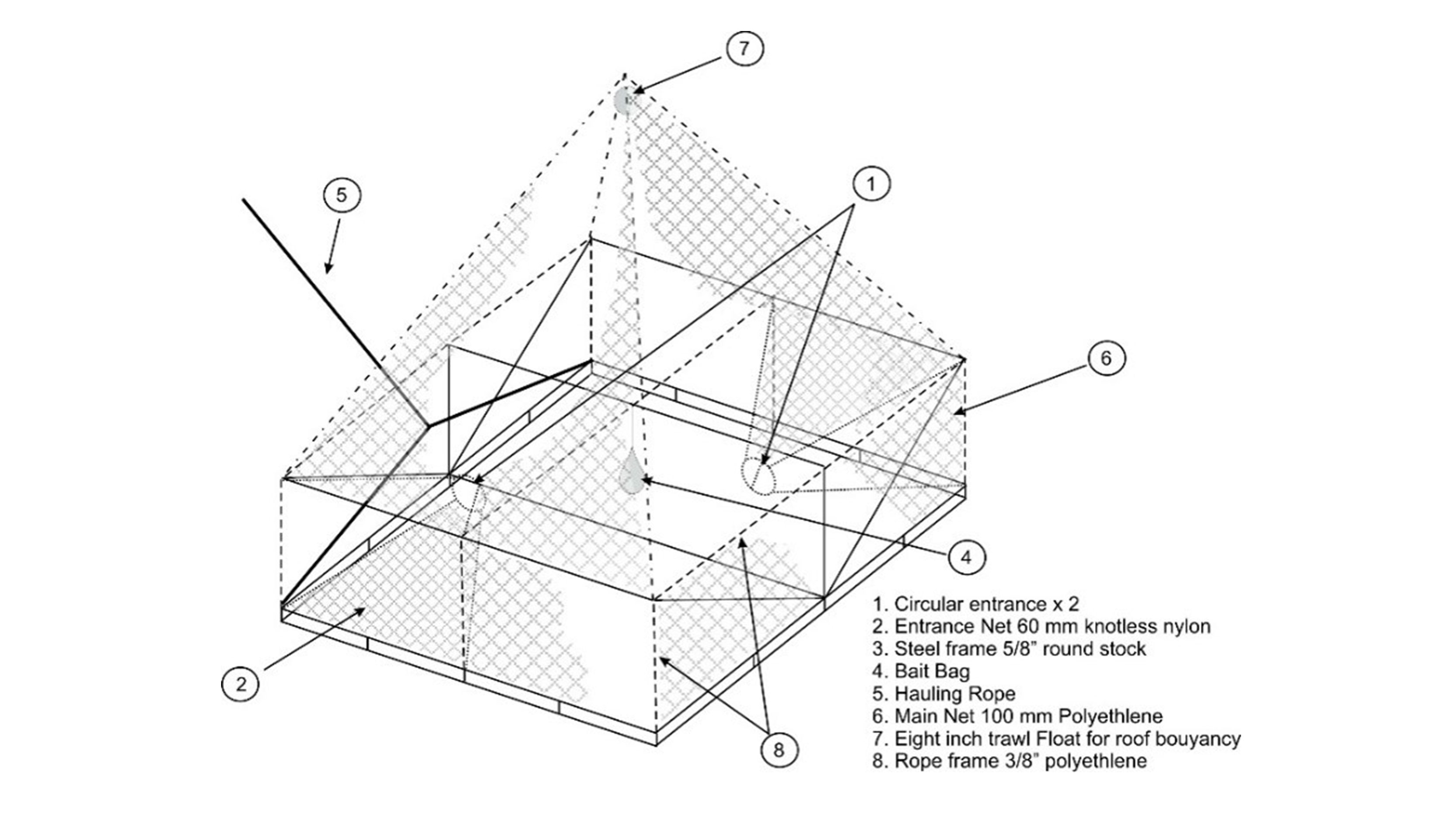

Supplement: Figure S1 — Diagram representing the Newfoundland (NL) cod pot used during our field research. [file peerj-05-2953-s004.png]
